# Sequential Acquisition of T Cells and Antibodies to Nontyphoidal *Salmonella* in Malawian Children

**DOI:** 10.1093/infdis/jiu045

**Published:** 2014-01-16

**Authors:** Tonney S. Nyirenda, James J. Gilchrist, Nicholas A. Feasey, Sarah J. Glennie, Naor Bar-Zeev, Melita A. Gordon, Calman A. MacLennan, Wilson L. Mandala, Robert S. Heyderman

**Affiliations:** 1 Malawi Liverpool Wellcome Trust Clinical Research Programme; 2 Biochemistry Section, Basic Medical Sciences Department, University of Malawi College of Medicine, Blantyre; 3 Department of Paediatrics, University of Oxford; 4 Liverpool School of Tropical Medicine; 5 Institute of Infection and Global Health, University of Liverpool; 6 Medical Research Council Centre for Immune Regulation, Institute of Biomedical Research, College of Medicine and Dental Sciences, University of Birmingham, United Kingdom; 7 Novartis Vaccines Institute for Global Health, Siena, Italy

**Keywords:** Salmonella, T cell, antibody, cytokine, children

## Abstract

***Background.*** *Salmonella* Typhimurium (STm) remain a prominent cause of bacteremia in sub-Saharan Africa. Complement-fixing antibodies to STm develop by 2 years of age. We hypothesized that STm-specific CD4^+^ T cells develop alongside this process.

***Methods.*** Eighty healthy Malawian children aged 0–60 months were recruited. STm-specific CD4^+^ T cells producing interferon γ, tumor necrosis factor α, and interleukin 2 were quantified using intracellular cytokine staining. Antibodies to STm were measured by serum bactericidal activity (SBA) assay, and anti-STm immunoglobulin G antibodies by enzyme-linked immunosorbent assay.

***Results.*** Between 2006 and 2011, STm bacteremias were detected in 449 children <5 years old. STm-specific CD4^+^ T cells were acquired in infancy, peaked at 14 months, and then declined. STm-specific SBA was detectable in newborns, declined in the first 8 months, and then increased to a peak at age 35 months. Acquisition of SBA correlated with acquisition of anti–STm–lipopolysaccharide (LPS) immunoglobulin G (*r* = 0.329 [95% confidence interval, .552–.062]; *P* = .01) but not anti–STm–outer membrane protein or anti–STm-flagellar protein (FliC).

***Conclusions.*** Acquisition of STm-specific CD4^+^ T cells in early childhood is consistent with early exposure to STm or cross-reactive protein antigens priming this T-cell development. STm-specific CD4^+^ T cells seem insufficient to protect against invasive nontyphoidal *Salmonella* disease, but sequential acquisition of SBA to STm LPS is associated with a decline in its incidence.

Nontyphoidal *Salmonella* (NTS), mainly *Salmonella enterica* serovars Typhimurium (STm) and Enteritidis, commonly causes bacteremia among young children in sub-Saharan Africa [1, 2]. Although NTS bacteremia is undergoing considerable epidemiological change [3, 4], the case fatality in children continues to exceed 20% [1]. Important risk factors for NTS bacteremia include age <2 years, malnutrition, severe malarial anemia, and human immunodeficiency virus (HIV) infection [1]. In resource-poor settings, lack of diagnostic services, increasing multidrug resistance, and the nonspecific nature of clinical presentation all compromise effective diagnosis and treatment of these children [1].

Evidence from whole-genome sequencing of STm, the most common NTS serovar isolated in Malawi, suggests that a pathovar characterized by multilocus sequence type 313 dominates invasive NTS (iNTS) disease in Africa [5]. Rarely seen in industrialized countries, sequence type 313 has undergone genomic degradation which suggests both the loss of an enteric lifestyle and possible human-host adaptation [6, 7]. Mouse models of disease caused by this facultative intracellular pathogen implicate innate immune cell phagocytosis, T-cell immunity, and antibody-mediated immunity [8, 9]. If iNTS is to be controlled effectively through public health interventions or vaccination, human studies are needed to establish the key immune components that constitute naturally acquired immunity in young children.

Most Malawian children acquire anti-*Salmonella* immunoglobulin G (IgG) and immunoglobulin M antibody and bactericidal activity against NTS by 2 years of age [10]. Antibodies targeting NTS can effect bacterial killing through activation of complement cascade and assembly of the membrane attack complex [10]. Antibodies opsonize NTS and, together with C3b deposition, facilitate internalization by phagocytes and subsequent killing of NTS through oxidative burst [11]. These immune processes are critical for preventing extracellular growth and dissemination of NTS [10]. Although it is known that CD4^+^ T cells orchestrate macrophage effector functions through interferon (IFN) γ and tumor necrosis factor (TNF) α [12, 13] and that HIV-infected individuals with low CD4 counts are particularly susceptible to iNTS disease [14], the contribution of CD4^+^ T-cell–mediated control of NTS in humans has not been well studied. We therefore explored the hypothesis that in the first 2 years of life CD4^+^ T-cell immune responses to STm develop in parallel with the development of anti-STm antibodies. Contrary to our expectations, we have found that although acquisition of STm-specific CD4^+^ T-cell immunity occurs together with antibody to STm protein antigens, these are evident before the development of serum bactericidal activity. This STm-specific CD4^+^ T-cell immunity seems insufficient to protect against iNTS disease in Malawian children, which declines in incidence in parallel with the later development of antibodies targeting STm LPS O-antigen.

## METHODS

### Setting and Bloodstream Infection Surveillance

Queen Elizabeth Central Hospital is a 1250-bed teaching hospital and the largest government hospital in Malawi, providing free health care to Blantyre district (population approximately 1 million). It is the only inpatient pediatric facility for non–fee-paying patients in Blantyre. The Malawi-Liverpool-Wellcome Trust Clinical Research Programme has undertaken routine bloodstream infection surveillance of febrile children presenting to Queen Elizabeth Central Hospital since 1997. Blood cultures are obtained from febrile children whose thick films are negative for malaria parasites or who are critically ill, irrespective of malaria infection. Blood culture is undertaken using a pediatric bottle (BacT/Alert PF BioMerieux), and isolates identified using standard techniques [15].

### Healthy Study Participants

A total of 80 healthy children (Table 1), in 8 predefined age categories ranging from 0 to 60 months, were prospectively recruited at a large community health center in Blantyre, Malawi, from March 2009 to January 2011. Children with malaria parasitemia, a positive HIV antibody test, severe anemia (hemoglobin <7 g/dL), malnutrition (weight-for-height *z* score ≤2), or other chronic illness were excluded from the study. Ethical approval for the study (protocol P.08/09/815) was obtained from College of Medicine Research Ethics Committee, and written informed consent was obtained from the parent or guardian of every participating child.

**Table 1.** General Characteristics and Nutritional and Hematological Profile

Parameter	Female Participants	Male Participants	All Participants
Participants, No. (%)	35 (43.7)	45 (56.3)	80 (100)
Age, median (range), mo	13.2 (0–52.5)	10 (0–47)	10.2 (0–52.5)
Weight, median (range), kg	9.5 (3.5–17)^a^	10 (6–16.9)^b^	ND
Height, median (range), cm	73.5 (48–97)^a^	74 (52–95)^b^	ND
Weight for height *z* score, median (range)	0.89 (−1.9–4.6)^a^	1.4 (−2–4)^b^	ND
Lymphocyte count, median (range), ×10^3^/μL	6.3 (2.9–13.46)	5.3 (2.2–10.4)	5.4 (2.2–13.6)
Hemoglobin, median (range), g/dL	11.5 (7.6–18.1)	11.2 (8.0–17.7)	11.4 (7.6–18.1)

Abbreviation: ND, not determined.

^a^ Twenty-five children aged 1-60 months were included.

^b^ Thirty-five children aged 1-60 months were included.

### Characterization of CD4^+^ Memory T-Cell Subsets

Whole blood was collected in ethylenediaminetetraacetic acid–anticoagulated tubes; 200 µL of blood was stained with antibodies (CD3 –allophycocyanin (APC), CD4-Pacific Blue, CD45RO–fluorescein isothiocyanate, and CCR7-phycoerythrin [all Becton Dickson]) and red blood cells lysed with 2 mL of 1× fluorescence-activated cell sorting (FACS) lysing solution (Becton Dickson). Cells were washed with phosphate-buffered saline (PBS; Sigma Aldrich) and fixed in 200 μL of 1% formaldehyde/PBS. Up to 20 000 events on a CD4^+^ T-lymphocyte gate were acquired immediately with a CyAN ADP flow cytometer (Beckman Coulter) and analyzed using FlowJo software (version 7.6.5, Tree Star). Lymphocytes were gated by their forward scatter and side scatter characteristics. We defined naive T cells as CD4^+^CD45RO^−^CCR7^+^, effector memory (EM) T cells as CD4 ^+^ CD45RO ^+^ CCR7^−^, and central memory (CM) T cells as CD4^+^CD45RO^+^CCR7^+^.

### Detection of CD4^+^ T cells Producing Cytokines

After whole blood for intracellular cytokine staining assay was collected in sodium heparin tubes, 450 μL of blood was stimulated with 50 μL of a bead-beaten STm strain D23580 [16] at the final concentration of 1 μg/mL or phorbol 12-myristate 13-acetate (PMA) at 1 µg/mL and ionomycin at 10 µg/mL (all Sigma Aldrich), and costimulated with anti-CD28/49d (Becton Dickson) for 6 hours at 37°C. At 2 hours, intracellular cytokine release was inhibited with BD Golgi Stop (Becton Dickson), and 200-μL samples were lysed with 2 mL of 1× FACS lysing solution and then permeabilized with 500 μL of 1× permeabilizing solution (Becton Dickson). Cells were washed with PBS/0.5% bovine serum albumin (BSA) buffer (Sigma Aldrich) and stained with 3 μL of surface antibodies (CD3-APC cyanine 7 and CD4-PB) and 5 μL of intracellular cytokine antibodies (IFN-γ–phycoerythrin, TNF-α–fluorescein isothiocyanate, and interleukin (IL) 2–APC [all Becton Dickson]). Cells were fixed and events acquired as described above. CD3^+^CD4^+^ T cells producing IFN-γ, TNF-α, and IL-2 were defined as CD3^+^CD4^+^IFN-γ^+^, CD3^+^CD4^+^TNF-α^+^, and CD3^+^CD4^+^IL-2^+^. Further analysis for polyfunctional CD4^+^ T cells producing single, double, and triple cytokines were analyzed by Boolean gates using FlowJo software.

### Quantification of STm-Specific Serum Bactericidal Activity

Serum bactericidal activity (SBA) assays were performed as described elsewhere [10]. Briefly, serum or PBS was mixed with STm D23580 [5], adjusted to 1.0 × 10^6^ CFU/mL, and incubated at 37°C for 180 minutes. Test samples were serially diluted and plated in triplicate on Luria-Bertani agar. *Salmonella* colony counts were done after 24 hours of incubation, and results were reported as log_10_ change in NTS count (CFU/mL) from the baseline.

### Quantifying Anti-NTS IgG Antibody by Enzyme-Linked Immunosorbent Assay

Enzyme-linked immunosorbent assay plates (Nunc-Immuno) were coated overnight using 100 µL of carbonate-bicarbonate buffer (Sigma Aldrich) per well containing the following antigens adjusted to 5 µg/mL: STm-LPS (Alexis Biochemicals), STm–outer membrane protein (OMP) and STm-flagellar protein (FliC) (kind gift from Adam Cunningham and Ian Henderson [17]), and *Escherichia coli*–LPS 0127:B8 (Sigma Aldrich). Plates were washed with wash buffer (PBS plus 0.05% Tween 20) and blocked with 200 µL of blocking buffer (PBS plus 1% BSA) per well for 1 hour at 37°C. Test serum at 1:20 in dilution buffer (PBS plus 0.05% Tween 20 plus 1% BSA) was serially diluted 3-fold and incubated at 37°C for 1 hour. After washing, 100 µL of 1:2000 secondary goat anti-human IgG-AP antibodies (Southern Biotech) were added and incubated for 1 hour at 37°C. Finally, after washing, 100 µL of SigmaFast p-nitrophenyl phosphate substrate was added to each plate and read after 30 minutes with a Bio Tek reader ELx800 (Bio Tek Instruments) at 405 nm.

### Statistical Analyses

We distinguished phases of the immune response as follows. Nonlinear regression models were fit to data relating STm-specific T cells and SBA responses with age. The inflection points of the resultant curves were taken to represent the boundaries of qualitatively different phases of immune response. We call the first period before the boundary the *early response,* and the subsequent period the *late response*. The immune responses within these early and late periods were then modeled using linear regression. GraphPad Prism software (version 5.0) was used to generate graphs and analyze the data.

## RESULTS

### Age Distribution of STm Bloodstream Infection in Children <5 Years Old in Malawi

**Figure 1.**
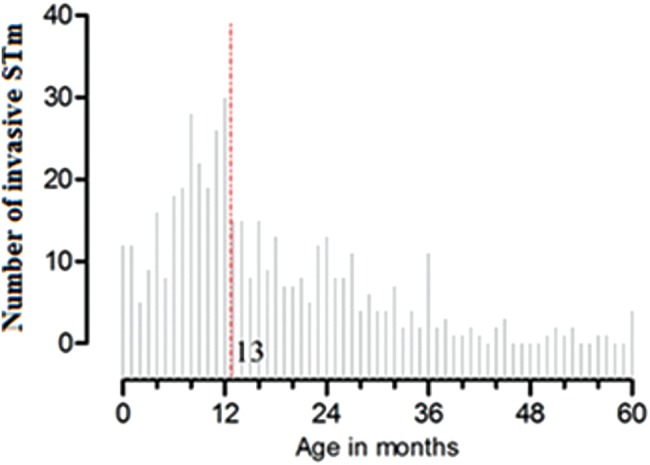
Age distribution of *Salmonella* Typhimurium (STm) bacteremia in children <5 years old at Queen Elizabeth Central Hospital, Blantyre, Malawi, 2006–2011 (N = 449); dashed line represents median age (13 months).

Between January 2006 and December 2011, STm bacteremia was detected in 449 children <5 years of age presenting to Queen Elizabeth Central Hospital, of whom 359 (80%) were <2 years old. The median age at STm bloodstream infection was 13 months (range, 0–60 months; Figure 1).

### Development of Memory CD4^+^ T-Cell Subsets in Children <5 Years Old

**Figure 2.**
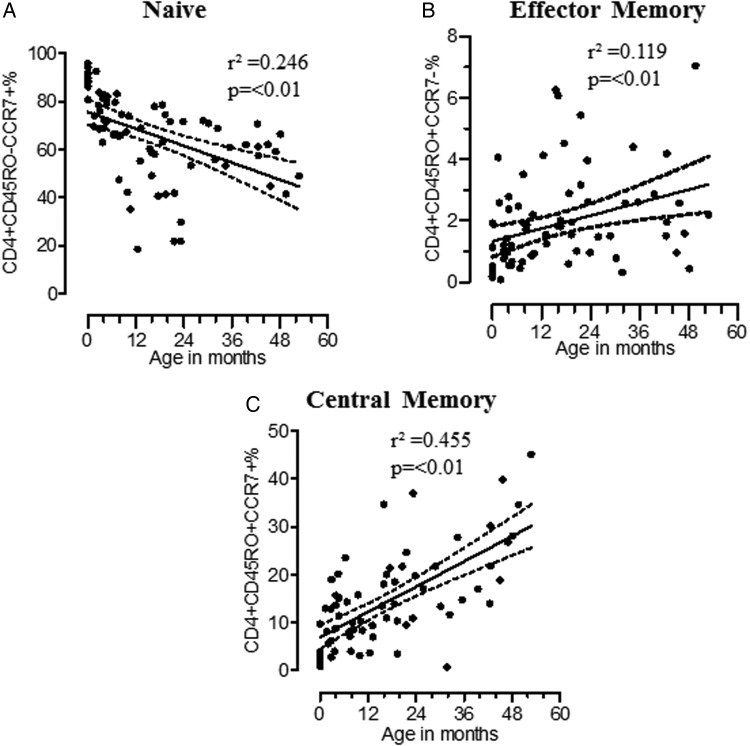
Development of memory CD4^+^ T-cell subsets in the first 5 years of life. Percentage are shown of naive CD4^+^ T cells: CD4^+^CD45RO^−^CCR7^−^ (*A*; n = 73), effector memory CD4^+^ T cells: CD4^+^CD45RO^+^CCR7^−^ (*B*; n = 73), and central memory CD4^+^ T cells: CD4^+^CD45RO^+^CCR7^+^) (*C*; n = 73) were plotted against age. Memory CD4^+^ T cells were determined by linear regression, represented by solid central lines, and 95% confidence intervals are represented by dashed lines.

To provide a context for the subsequent assessment of functional T-cell memory, we first assessed the overall development of T-cell subsets in this Malawian population. Newborns are pathogen inexperienced [18], and therefore CD4^+^ T cells develop memory with age, enabling them to mount rapid immune responses to previously encountered pathogens. Naive, EM, and CM CD4^+^ T cells can be differentiated by their extracellular expression of CD45RO and CCR7 [19, 20]. As expected [21], we found that the proportion of CD4^+^CD45RO^−^CCR7^+^ naive T cells decreased with age (*r*² = 0.246; slope, −0.58 [95% confidence interval (CI), −.83 to −.34]; *P* ≤ .01; Figure 2*A*). The proportion of CD4^+^CD45RO^+^CCR7^−^ EM (*r*² = 0.119; slope, 0.035 [95% CI, .012–.057]; *P* ≤ .01) and CD4^+^CD45RO^+^CCR7^+^ CM (*r*² = 0.455; slope, 0.43 [95% CI, .32–.55]; *P* ≤ .01) T cells increased with age (Figure 2*B* and 2*C*).

### Early Acquisition of STm-Specific CD4^+^ T-Cell Immune Responses

**Figure 3.**
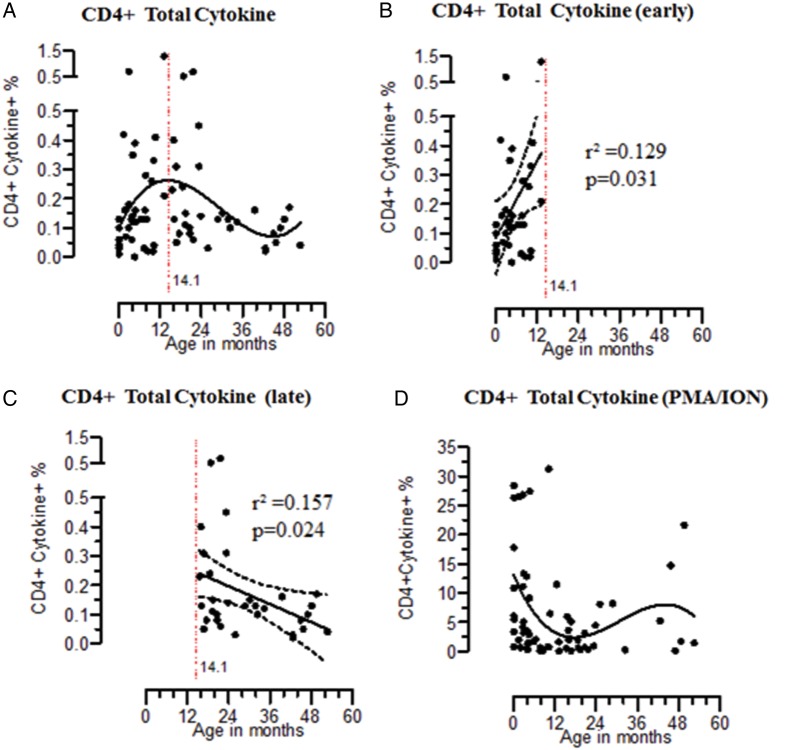
Early acquisition of *Salmonella* Typhimurium (STm)–specific CD4^+^ T-cell immune responses. Percentage are shown for STm-specific CD4^+^ T cells producing total (*A*; n = 68), early (*B*; n = 36), and late (*C*; n = 32) cytokine and phorbol 12-myristate 13-acetate (PMA)/ionomycin stimulated CD4^+^ T cells producing total cytokine (*D*; n = 62). Nonlinear polynomial regression models of third order were fit to data relating specific T-cell cytokine response to age. STm-specific T cells response within early and late periods was determined by linear regression, represented by solid central lines; dashed lines represent 95% confidence intervals.

We next sought to explore the hypothesis that CD4^+^ T-cell immune responses to STm develop in parallel with acquisition of antibody-mediated immunity. Contrary to our hypothesis, we found that STm-specific CD4^+^ T cells producing cytokines were present early in life, peaked at 14 months and then declined (Figure 3*A*). This was further analyzed by using the nonlinear model peak points to define early and late STm-specific CD4^+^ T cells. This showed early acquisition of STm-specific CD4^+^ T-cell immunity (*r*² = 0.129; slope, 0.021 [95% CI, .002–.041]; *P* = .031), followed by a decrease in older children (*r*² = 0.157; slope, −0.005 [95% CI, −.009 to −.0006]; *P* = .024; Figure 3*B* and 3*C*). These changes in intracellular cytokine profiles mirrored changes in IFN-γ– and TNF-α– rather than IL-2–secreting cells (see Supplementary Figure 1). STm-specific CD4^+^ cytokine responses did not correlate with PMA-stimulated CD4^+^ T-cell cytokine responses (*r* = 0.109 [95% CI, −.128 to .371]; *P* = .426; Table 2), indicating that these responses to STm antigens were not simply due to a general maturation of the immune system (Figure 3*D* and Supplementary Figure 2). Generation of antigen-specific multiple cytokine-producing cells is widely thought to indicate maturation of antigen-specific CD4^+^ T-cell responses [22]. Maturation of STm-specific T-cell responses in these healthy children (either double or triple cytokine producers) peaked mostly between 13–24 months and subsequently declined, whereas for IL-2^+^TNF-α^+^CD4 T cells, the response was sustained (data not shown).

**Table 2.** Association Between Immune Variables

Parameter (s)	XY Pairs	Spearman *r*	95% CI	*P* Value
NTS vs PMA CD4^+^ cytokine^+^	55	0.109	−.128 to .371	.426
SBA vs anti–STm-LPS IgG antibody titers	55	0.329	.552–.062	.01
SBA vs anti–STm-OMP IgG antibody titers	57	0.044	−.226 to .308	.741
SBA vs anti–STm-FliC IgG antibody titers	58	−0.001	−.266 to .264	.992
SBA vs anti–*E. coli*-LPS IgG antibody titers	50	0.031	−.257 to .314	.830
CD4^+^ cytokine^+^ vs anti–STm-OMP IgG antibody titers	65	0.137	−.117 to .375	.275
CD4^+^ cytokine^+^ vs anti–STm-FliC IgG antibody titers	67	0.174	−.075 to .404	.157
CD4^+^ cytokine^+^ vs anti–STm-OMP IgG antibody titers (early)^a^	39	0.405	.088–.647	.01
CD4^+^ cytokine^+^ vs anti–STm-FliC IgG antibody titers (early)^a^	38	0.394	.080–.637	.01
CD4^+^ cytokine^+^ vs anti–STm-LPS IgG antibody titers (early)^a^	36	−0.257	−.547 to .087	.129

Abbreviations: CI, confidence interval; *E. coli, Escherichia coli*; FliC, flagellar protein; IgG, immunoglobulin G; LPS, lipopolysaccharide; NTS, nontyphoidal *Salmonella*; OMP, outer membrane protein; PMA, phorbol 12-myristate 13-acetate; SBA, serum bactericidal activity; STm, *Salmonella* Typhimurium.

^a^ *Early* refers to parameters of participants aged <14 months.

### Delayed Acquisition of STm-Specific SBA

**Figure 4.**
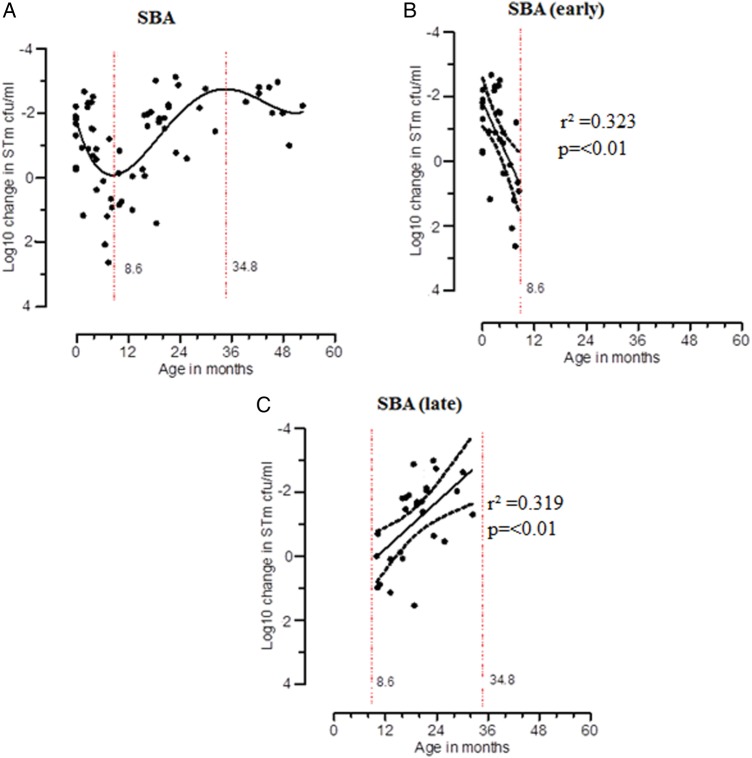
Acquisition of *Salmonella* Typhimurium (STm)–specific serum bactericidal activity (SBA) among children. The log_10_ change in STm (in colony-forming units [CFU] per milliliter) relative to the control condition was plotted against age. The y-axis was inverted. Nonlinear regression polynomial model is represented by solid lines (*A*; n = 65). SBA responses within early (*B*; n = 29) and late (*C*; n = 27) periods was determined by linear regression, represented by solid central lines; dashed lines represent 95% confidence intervals.

**Figure 5.**
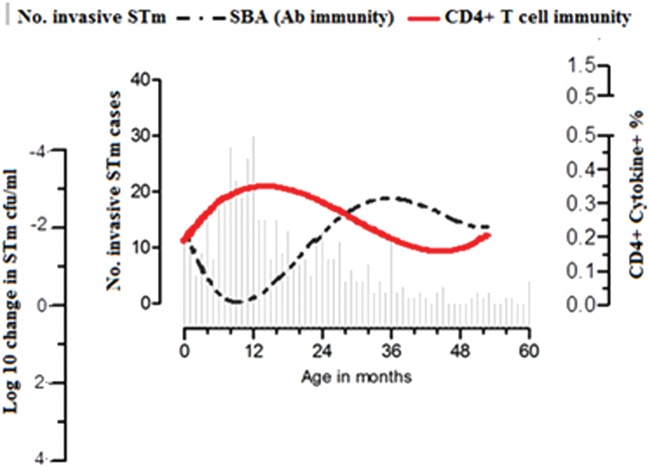
Sequential acquisition of T cells and antibodies to *Salmonella* Typhimurium (STm) in children. Age distribution of STm bloodstream infection in children <5 years old at Queen Elizabeth Central Hospital (Blantyre, Malawi; 2006–2011) was superimposed with kinetics of STm-specific CD4^+^ T-cell immune responses and STm-specific serum bactericidal activity (y-axis was inverted) in children aged 0–60 months. Abbreviations: CFU, colony-forming units; SBA, serum bactericidal activity.

To confirm previous observations made in Blantyre by MacLennan et al, we used the same SBA assay and clinical STm strain D23580 [10]. In line with the previous findings, STm-specific SBA declined in the first 8 months of life and then increased to a peak at 35 months (Figure 4*A*). To further analyze these trends in NTS-specific SBA, we divided the periods into early and late phases according to peak and nadir points, as before. We found that STm-specific SBA declined in the first 8 months of life (*r*² = 0.323; slope, 0.292 [95% CI, .125–.459]; *P* ≤ .01) and then increased between 8 and 35 months (*r*² = 0.319; slope, −0.121 [95% CI, −.193 to −.048]; *P* ≤ .01; Figure 4*B* and 4*C*). This STm-specific increase in SBA occurred later than that seen in T-cell immunity to STm (Figures 3–5).

### Correlation of STm-Specific SBA With Presence of Antibodies Targeting STm-LPS

Previous work in HIV-infected Malawian adults showed that excess anti-LPS IgG antibodies can inhibit complement-mediated killing of NTS in vitro, whereas antibodies to OMPs can mediate bactericidal activity [17]. To clarify the antigenic targets of the STm-specific antibody in children, we measured serum antibodies to STm LPS, OMP, FliC, and *E. coli* 0127:B8 LPS. We found that anti–STm-LPS IgG antibody titers mirrored the pattern seen with SBA assay (Figure 4*A* and Supplementary Figure 3*A*). Anti–STm-OMP antibody titers were lowest at birth and increasing with age, whereas anti–STm-FliC IgG and anti–*E. coli*–LPS IgG antibody titers showed no particular trend with age (Supplementary Figure 3*B*–3*D*). The correlation between SBA and anti–STm-LPS IgG titers (*r* = 0.329 [95% CI, .552–.062]; *P* = .01), and the lack of correlation between SBA and anti–*E. coli*-LPS titers suggests that SBA is due to STm-specific rather than nonspecific LPS antibodies (Table 2). A lack of a correlation with anti–STm-OMP and anti–STm-FliC suggest that these targets do not substantially contribute to SBA in these children.

### STm-Specific CD4^+^ T-Cell Immune Responses in Early Childhood Associated With Generation of Anti-STm Protein Antibodies

Having shown that STm-specific CD4^+^ T cells peak in early life (Figure 5), we investigated whether this immune memory was linked to the generation of anti–STm-OMP and anti–STm-FliC IgG antibodies. We found that STm-specific CD4^+^ T-cell immune responses correlate with anti–STm-OMP and anti–STm-FliC IgG antibodies in early childhood (*r* = 0.405 [95% CI, .088–.647; *P* = .01] and *r* = 0.394 [95% CI, .080–.637; *P* = .01], respectively) and not anti–STm-LPS IgG antibodies (*r* = −0.257 [95% CI, −.547 to .087]; *P* = .129; Table 2). This contemporaneous development of antibodies to STm OMP and T-cell immunity is in line with the conventional paradigm of the T-cell–dependent immune response to a protein antigen [23, 24].

## DISCUSSION

NTS infection in African children is associated with life-threatening bacteremia. Here we extend previous observations to show that STm-specific CD4^+^ T-cell immunity is acquired early in childhood in parallel with antibody to STm protein antigens but precedes the development of complement-fixing antibody immunity. These findings suggest exposure to STm or cross-reactive protein antigens induces STm-specific CD4^+^ T-cell immune responses early in life, presumably within the gut-associated lymphoid tissues [25]. Enteric pathogens colonize the gastrointestinal tract soon after birth, even in exclusively breastfed babies [26]. However, the incidence and frequency of *Salmonella* colonization of the gastrointestinal tract in this population, and whether repeated *Salmonella* infections are required to generate this natural immunity, is not known. Most Malawian children are initially exclusively breastfed but are then weaned onto mixed feeding after 3 months of age [27, 28]. This switch in food seem s to coincide with the observed emergence of STm-specific T cells and the appearance of anti–STm-OMP and anti–STm-FliC IgG antibodies. It is uncertain why STm-specific T-cell immunity declines in older children following evidence of immune maturation, but this could be due to decreased exposure of the immune system to STm and homing of residual specific CM CD4^+^ T-cell memory to lymphoid tissues. To what extent this T-cell and B-cell immunity protects against NTS then becomes a key question.

Both previous [10] and current surveillance in Malawi show that 80% of STm bacteremia cases occur in children <24 months among under five children, with a peak at 13 months. Acquisition of STm-specific CD4^+^ T cells in early childhood parallels the age-related increase in incidence of STm bacteremia, suggesting that the early acquisition of T-cell immunity to NTS alone is insufficient to protect against iNTS disease and that additional immune modalities are required. The association of an age-related decline in incidence of STm bacteremia with increasing levels of STm-specific complement-fixing antibodies is suggestive of protective immunity [10]. Indeed, both a previous study [10] and the current one found that STm-specific SBA is detectable in newborns (consistent with passively acquired maternal antibody) and that the natural decline in this antibody with age coincides with an increase in the incidence of iNTS.

The strong relationship between anti–STm-IgG antibodies targeting STm-LPS and SBA, and a lack of correlation with STm-OMP, STm-FliC, or *E. coli*–LPS support previous evidence that anti–STm-LPS IgG antibodies mediate this SBA [29–31] and suggest that these antibodies recognize the variable component of LPS (O-antigen)*.* In some HIV-infected adults with dysregulated humoral immunity and hypergammaglobulinemia, excess IgG antibody to STm-LPS prevents killing of NTS [17], but this is at levels much higher than those found in healthy HIV-uninfected children and adults and was not apparent in our studies.

Our findings do not preclude an important role for T cells in elimination of salmonellae from the intracellular niche. Clearance of disseminated *Salmonella* infection is thought to require a specific Th1 response [32]. Mastroeni [33] hypothesized, based on murine models, that protective immunity to *Salmonella* infection is acquired in a stepwise fashion constituting innate cells, T cells, and then antibody. Preexisting antibodies against *Salmonella* reduce murine bacteremia by preventing early infection [34]. Protection induced by heat-killed salmonellae correlates with anti-*Salmonella* antibody titers [35], with SBA attributable to anti-LPS antibodies [29] and with binding of *Salmonella*-specific antibodies. These facilitate the development of T-cell immunity by enhancing bacterial uptake through opsonization and also antigen presentation by macrophages [36].

Based on our human studies, it is likely that the early development of T cells specific for STm protein antigens and subsequent cognate interactions with B cells leads to antibody production against these antigens, class-switching, affinity maturation and memory formation [23]. We speculate that, in view of the complex nature of the *Salmonella* antigens presented during natural exposure, these STm protein-specific T cells may also provide bystander (hapten-carrier) help to B cells specific for STm-LPS. LPS alone is a T-cell–independent type 2 antigen, but when taken up by antigen presenting cells in combination with STm proteins, has potential to act in the same way that polysaccharide-conjugate vaccines generate isotype-switched memory B-cell immunity [37].

In conclusion, STm-specific CD4^+^ T cells seem insufficient to protect against iNTS disease, but sequential acquisition of SBA to STm LPS is associated with a decline in incidence of iNTS. STm-specific CD4^+^ T cells may drive the development of protective antibody responses through bystander interactions with B cells. Given the burden of iNTS in sub-Saharan Africa [2], a vaccine is urgently required. STm LPS O-antigen has considerable potential as a vaccine target, and there are currently several groups developing conjugate vaccines for this purpose to overcome the short-lived T-independent antibody response generated by polysaccharide alone [38]. Immunization with STm-OMP and STm-FliC induce both T cells and antibodies in animal models and are therefore also being investigated as vaccine candidates, either separately [39] or covalently linked to O-antigen as glycoconjugates [40].

## Supplementary Data

Supplementary materials are available at *The Journal of Infectious Diseases* online (http://jid.oxfordjournals.org/). Supplementary materials consist of data provided by the author that are published to benefit the reader. The posted materials are not copyedited. The contents of all supplementary data are the sole responsibility of the authors. Questions or messages regarding errors should be addressed to the author.

Supplementary Data
