# Supplementary material for: Sequential Acquisition of T Cells and Antibodies to Nontyphoidal Salmonella in Malawian Children
Source: J Infect Dis. 2014 Jan 16;210(1):56–64. doi: 10.1093/infdis/jiu045 (PMC4054899; doi:10.1093/infdis/jiu045)
Supplement: Supplementary Data [file supp_210_1_56__index.html]

Sequential acquisition of T-cells and antibodies to nontyphoidal Salmonella in Malawian Children — Sequential Acquisition of T Cells and Antibodies to Nontyphoidal Salmonella in Malawian Children — Sequential Acquisition of T Cells and Antibodies to Nontyphoidal Salmonella in Malawian Children — Supplementary Data 

# Sequential Acquisition of T Cells and Antibodies to Nontyphoidal *Salmonella* in Malawian Children

## Supplementary Data

Supplementary Data

**Files in this Data Supplement:**

- Supplementary Data - Doc file
- Supplementary Figure 1 - tif file
- Supplementary Figure 2 - tif file
- Supplementary Figure 3 - tif file
