# Supplementary material for: Sequential Acquisition of T Cells and Antibodies to Nontyphoidal Salmonella in Malawian Children
Source: J Infect Dis. 2014 Jan 16;210(1):56–64. doi: 10.1093/infdis/jiu045 (PMC4054899; doi:10.1093/infdis/jiu045)
Supplement: Supplementary Data [file supp_jiu045_jiu045supp.doc]

**Supplementary Figure 1: Early acquisition of STm-specific CD4+T cell immune responses**

Blood samples were analyzed for STm-specific CD4+ T cells producing cytokines using ICS assay. Percentage of STm-specific CD4+ T cells producing IFN-γ (Fig 1a, n=68), IL-2 (Fig 1b, n=68), TNF-α (Fig 1c, n=67). The immune response with age was determined by nonlinear regression polynomial models represented by solid line.

**Supplementary Figure 2: Acquisition of PMA/ION-specific CD4+T cell immune responses**

Blood samples were analyzed for PMA/ION specific CD4+ T cells producing cytokines using ICS assay. Percentage of PMA/ION-specific CD4+ T cells IFN-γ (Fig 2a, n=62), IL-2 (Fig. 2b, n=62), TNF-
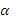
 (Fig. 2c, n=62). The immune response with age was determined by nonlinear regression polynomial models represented by solid line.

**Supplementary Figure 3**: **Age dependent acquisition of specific anti-STm-IgG antibodies** Serum from children was tested for anti-STm-specific IgG antibodies using ELISA. Anti-STm-LPS IgG (Fig. 3a, n=63), anti-STm OMP IgG (Fig. 3b, n=66), anti-STm-FliC IgG (Fig. 3c, n=67) and anti-*E.Coli*-LPS IgG antibody titers (Fig. 3d, n=63) were plotted against age. SBA with age was determined by nonlinear regression polynomial models represented by solid line.
