# Supplementary figures and images for: Sequential Acquisition of T Cells and Antibodies to Nontyphoidal Salmonella in Malawian Children
Source: J Infect Dis. 2014 Jan 16;210(1):56–64. doi: 10.1093/infdis/jiu045 (PMC4054899; doi:10.1093/infdis/jiu045)

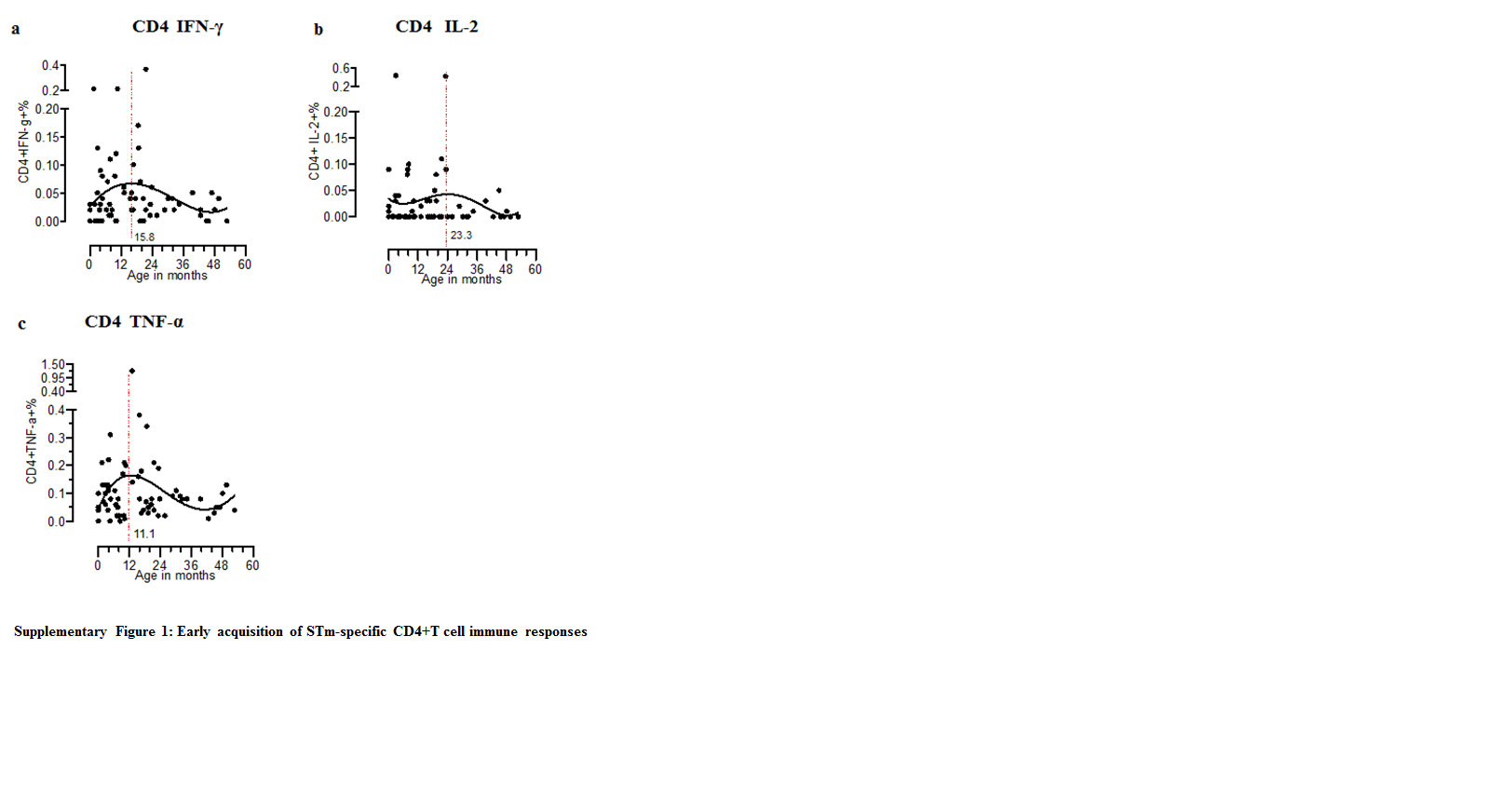

Supplement: Supplementary Data [file supp_jiu045_jiu045supp_fig1.tif]

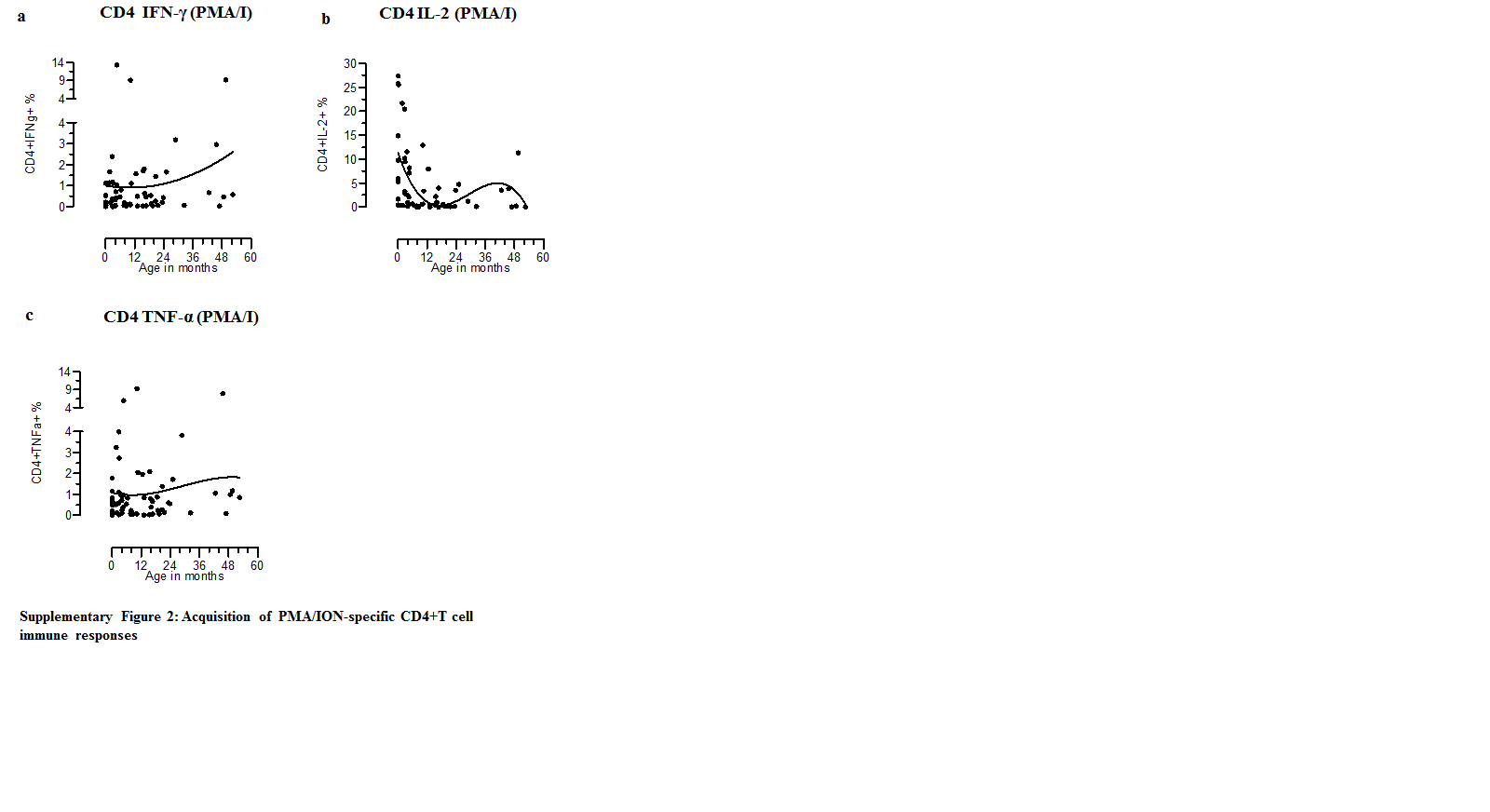

Supplement: Supplementary Data [file supp_jiu045_jiu045supp_fig2.tif]

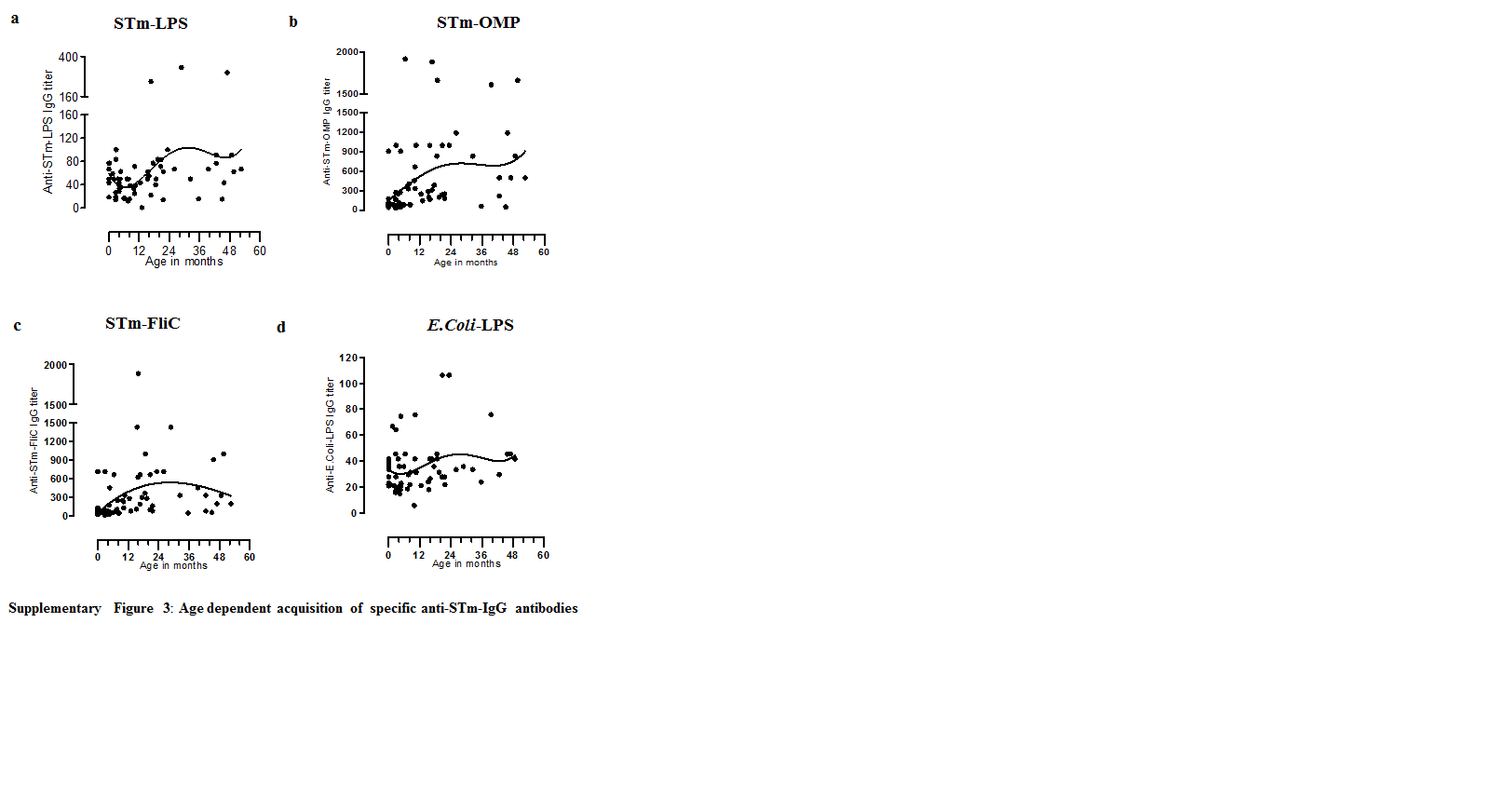

Supplement: Supplementary Data [file supp_jiu045_jiu045supp_fig3.tif]
